# Can hook-bending be let off the hook? Bending/unbending of pliant tools by cockatoos

**DOI:** 10.1098/rspb.2017.1026

**Published:** 2017-09-06

**Authors:** I. B. Laumer, T. Bugnyar, S. A. Reber, A. M. I. Auersperg

**Affiliations:** 1 Department of Cognitive Biology, University of Vienna, Althanstr. 14, 1090 Vienna, Austria; 2 Messerli Research Institute, University of Veterinary Medicine (other partner institutions: University of Vienna, Medical University of Vienna), Veterinärplatz 1, 1210 Vienna, Austria

**Keywords:** tool use, tool manufacture, innovation, problem solving, avian cognition

## Abstract

The spontaneous crafting of hook-tools from bendable material to lift a basket out of a vertical tube in corvids has widely been used as one of the prime examples of animal tool innovation. However, it was recently suggested that the animals' solution was hardly innovative but strongly influenced by predispositions from habitual tool use and nest building. We tested Goffin's cockatoo, which is neither a specialized tool user nor a nest builder, on a similar task set-up. Three birds individually learned to bend hook tools from straight wire to retrieve food from vertical tubes and four subjects unbent wire to retrieve food from horizontal tubes. Pre-experience with ready-made hooks had some effect but was not necessary for success. Our results indicate that the ability to represent and manufacture tools according to a current need does not require genetically hardwired behavioural routines, but can indeed arise innovatively from domain general cognitive processing.

## 1. Introduction

In 2002, the New Caledonian crow (*Corvus moneduloides*) ‘Betty’ spontaneously bent a straight piece of wire into a hook to lift a basket out of a vertical tube, after her mate had flown off with the appropriate tool [1]. This was long considered one of the most important textbook examples for innovative tool modification in animals, as Betty fashioned her tool from a novel (pliant) material and was thought to lack previous experience in hook bending when she was still in the wild [1–7]. Follow-up experiments even suggested context-dependent flexibility within her interaction with the material, as Betty bent and unbent novel material using different techniques [8]. Unbending (straightening) of materials was also investigated in habitually tool using primates [9,10]. Due to the material chosen and the strength of primates it is, however, doubtful whether success was intentional [8]. Although, wild New Caledonian crows are the only animals that have been observed crafting hooks into the wide ends of branches, the natural hook crafting behaviour that was observed at the time did not involve any bending manipulations of the material [11,12].

Interestingly this type of tool innovation was later also tested in human infants and was found to develop surprisingly late: children failed the task up to the age of five and it was not until 8 years of age that the majority of subjects successfully bent the wire [13,14]. At the time, the authors suggested that hook bending may represent an ‘ill-structured’ problem as subjects only have information about the start and goal states but not on how to get from one state to another and must therefore learn to amplify relevant actions and inhibit irrelevant actions while keeping the solution in mind [14].

Yet, the interpretation of Betty's hook bending recently came under scrutiny after studies on wild-caught New Caledonian crows revealed that the bending and unbending of natural tool material seems to be part of the species natural behavioural routine [15,16]. Most of their subjects carefully bent the end of their tools, supposedly to add more curvature to the tool shaft, using techniques closely resembling those used by Betty when bending or unbending wire. As it is hard to retrace innovation events in habitual tool users, it is almost impossible to distinguish tool innovations that have spread throughout a population from heritable behavioural patterns. At the moment, it is unclear to what extent genetically hardwired behaviour, sophisticated cognition, or a mixture of both account for Betty's behaviour. A presently more convincing example was provided by rooks solving the hook bending problem after previous experience with ready-made hooks [17]. As rooks are not specialized tool users their behaviour cannot be evoked from inherited routines from habitual tool manufacture as has been argued for New Caledonian crows [15,16]. Nevertheless, it was suggested that their success could still be influenced by behavioural predispositions, such as yanking and twisting actions required during nest construction [16]. Rook nests (like those of many corvids) consist of thin, freshly broken twigs that appear to be more strongly bent at the centre of the nest (AMI Auersperg personal observation).

Goffin's cockatoos (*Cacatua goffiniana*) do not show morphological or behavioural adaptations for using tools, nor do they seem to habitually use tools in the wild: our research group has been looking specifically into their foraging behaviour in their original habitat (Tanimbar/Moluccas) for several months (more than 1800 individual recordings) and has additionally been studying a wild caught group (*n* = 20) without being able to record any of the above behaviours. Despite a single observation of a bird from an introduced population in Singapore combining a flower stalk with a coconut (A Osuna-Mascaro, AMI Auersperg 2017, unpublished data) we lack any evidence of possible tool-related behaviour in wild Goffin's cockatoos. Furthermore, all Cacatuidae use pre-existing cavities for nesting (some species scrape the inside of their holes with their beaks and some add some soft materials to the floor of the cavity but do not construct complex nest constructs out of sticks or woven nests that require the establishment of complex object relationships or the bending of materials) [18]. Goffin's cockatoos breed in pre-existing tree holes and do not seem to modify their cavities; our field-team has been able to inspect seven nests so far (M O'Hara 2017, personal communication). Nevertheless, Goffin's cockatoos have shown the capacity to innovatively manufacture and use both compact and stick-type tools under laboratory conditions [19–22]. These animals are opportunist island birds that feed on various, sometimes seasonal or new resources that often require different extractive foraging techniques (ongoing field research; B Mioduszewska, M O'Hara, DM Prawiradilaga, L Huber, AMI Auersperg, unpublished data). Their problem-solving and tool using abilities are thus unlikely to be influenced by behavioural predispositions but arise from a combination of general flexibility and powerful learning abilities [23]. This assumption points the way towards the experiment reported here, in which we pursued two main aims (i) We wanted to investigate whether innovative tool bending or unbending can arise in a bird species that apparently lacks any ecological pre-dispositions for bending a material during tool-related foraging and nest building. For this, we applied a similar set-up as previously used for corvids, providing the birds with vertical and horizontal tubes that were too long to reach the food inside and offering materials that required bending/unbending to work as functional tools [8]. (ii) We aimed to identify the effect of individual pre-experience with hooks for potential task success. For this we divided birds into two groups, with one group receiving stepwise scaffolding of experience and the other group serving as the control group, receiving a similar amount of opportunities to manipulate the apparatus but no scaffolding steps.

If cockatoos are quick learners with good generalization skills, they should profit from scaffolding, i.e. birds from the group with pre-experience should do better than birds from the control group. If cockatoos innovate solutions to this novel problem, some individuals might discover toolmaking independently of their scaffolding history.

## 2. Methods

### (a) Subjects

Five female and eight male adult, captive-reared Goffin's cockatoos participated. All had experience in making straight stick-type tools but had no experience with pliant materials or the use of hooks prior to this experiment (for detailed individual information and experimental histories see electronic supplementary material, section A, table S1).

While all subject species previously tested on hook bending had pre-experience with ready-made hooks [1,8,13,14,17] the cockatoos in our captive colony were naive in respect to any pliant materials and the use of hooks at the onset of the study.

### (b) Apparatuses and procedures

**Figure 1.**
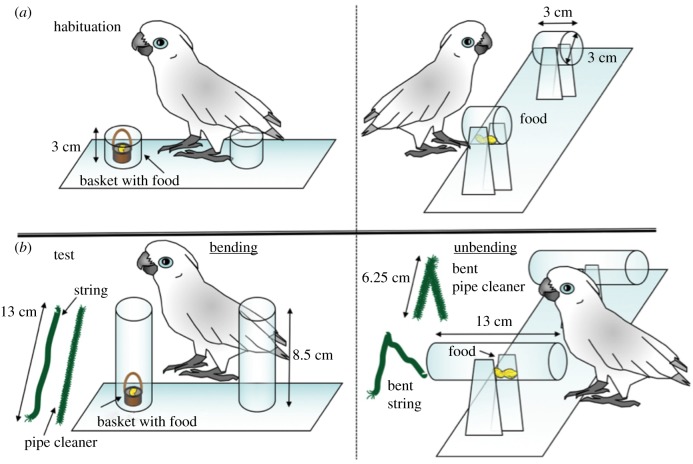
(*a*) Apparatuses for the habituation phase; (*b*) apparatuses for the testing phase. Left: vertical tube apparatus; right: horizontal tube apparatus. Dimension are given in centimetres.

Previous to the actual experiment, the birds were habituated to the apparatus and the materials separately. They were allowed to interact with an apparatus with shortened tubes so they could reach the food (see description of main apparatus below and figure 1*a*) and to observe an experimenter wrapping the material around a pen to give subjects the information that the wire but not the string retains its form after bending (for details of the habituation procedure see electronic supplementary material, section B).

All subjects were exposed to a ‘bending’ and an ‘unbending’ task. In both tasks the testing apparatus consisted of two Plexiglas tubes with one tube being baited with a piece of cashew nut. The tubes were vertical for the bending task and horizontal for the unbending task and in both cases too long for the birds to directly reach the reward (figure 1*b*). Apparatuses were always baited out of sight. Two materials, green string and green pipe-cleaner (straight in the bending task and bent at 45° in the unbending task) were offered on both sides of the set-up. For all tasks the position of the food reward, as well as the wire and string, were semi-randomly balanced across sessions.

Subjects were divided into two groups, with group E (experience group) being the test group that received scaffolding pre-experience and group C being a control group that also received a similar amount of opportunities to manipulate the apparatus but no pre-experiences (see electronic supplementary material, section A). For each group the order in which subjects received *Phase I* of either the bending or the unbending task was counterbalanced.

Testing was conducted in three phases (*Phase I–III*, further explained below). A session consisted of up to 10 trials. Subjects were tested for 15 min per trial or until success. A trial started as soon as the subject touched a material and lasted either for 15 min elapsed or until task success. If the subject did not interact with the materials for 15 min the trial was terminated. If the subject was not successful within a trial, the session was ended. If a subject was successful it received up to nine additional trials. If a subject was continuously successful for 10 trials within one session it received up to 10 trials on the subsequent test day. Testing was continued until subjects were successful a total of 20 trials within two out of three consecutive sessions or until completing *Phase III* without being successful within three consecutive test days.

**Figure 2.**
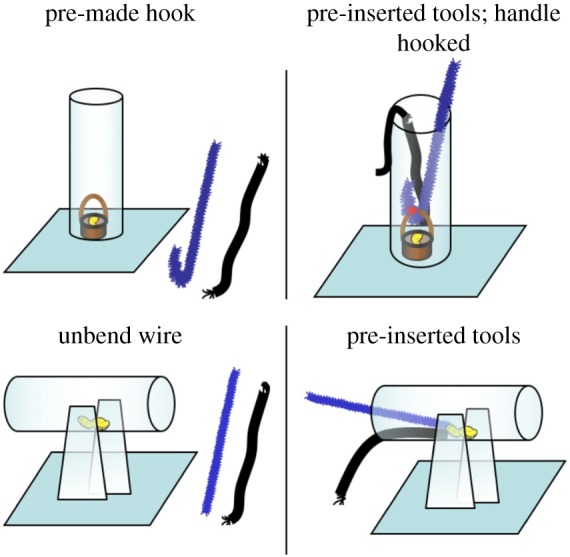
(*a*) *Pre-experience A* in the vertical (above; wire bent to hook) and horizontal condition (below; wire straightened). (*b*) *Pre-experience* B in the vertical (above; pre-bent hook and string inserted) and horizontal condition (below; straightened wire and string inserted).

During *Phase I (Naive phase)* both groups received one session of each task (bending & unbending) without pre-experience. During *Phase II* (*Pre-experience A*) only Group E was supplied with a premade hook tool (bending task, vertical tubes) or a ready-made straight tool (unbending task, horizontal tubes) and a same shaped string alongside the apparatus (figure 2). If a subject was successful in the first trial it received up to nine additional trials of the respective pre-experience. If continuously successful during 10 *Phase II* trials, subjects received a *Phase I* trial. If not successful in *Phase I*, the birds received only three trials of *Phase II* followed by a *Phase I* trial in the subsequent sessions. If not successful in this *Phase I* trial, the sequence was repeated in the following test sessions until reaching Phase *III (see overview* electronic supplementary material, *section A,* figures S1 and S2)*.* Subjects that were successful in the *Phase I* trial received up to nine additional *Phase I* trials if continuously successful. Testing was then continued until subjects were successful a total of 20 *Phase I* trials within two out of three consecutive sessions. The control group was tested as in *Phase 1 (Naive phase)*. All subjects (also those that were never successful in any *Phase I* trial) were tested for five sessions in *Phase II and Phase III*.

In *Phase III* (Pre-experience B) subjects in group E faced a set-up in which the pre-made tool and the string were pre-inserted into the apparatus (in the bending task the hook was hinged into the handle of the reward basket inside the vertical tube; see figure 2). If 10 times successful within one session, subjects were subsequently tested as in *Phase I*. If not successful in *Phase I*, subjects received from then on in the subsequent sessions only three trials of *Phase III* followed by a *Phase I* trial. Subjects that were successful in the *Phase I* trial received up to nine additional *Phase I* trials if continuously successful. Testing was then continued until subjects were successful a total of 20 *Phase I* trials within two out of three consecutive sessions. After receiving five sessions of *Phase II and* five sessions of *Phase III* testing was continued until the subject was unsuccessful in *Phase 1* for three consecutive sessions. The control group was tested as in *Phase I* (*Naive phase*).

All trials were HD video recorded and after each session the bent wires were photographed and stored. During testing the experimenter was present behind the camera but wore mirrored sunglasses, avoided any head movements and was not speaking.

### (c) Analysis

For the video coding we used BORIS [24]. We checked inter-observer reliability (12% of the videos were double coded) and found excellent agreement (ICC ≥ 0.819; *p* < 0.001). In order to maintain comparability to the corvid studies, we recorded and analysed the same variables as Weir & Kacelnik [8]: ‘time until success’, ‘latency between start of the trial and the first modification of the wire’, ‘duration of probing with the functional end of the modified tool’, ‘duration of probing with the unmodified wire’, ‘duration of probing with the modified non-functional tool’, and ‘tool crafting time’ (see electronic supplementary material, section B, table S2 for descriptions of all variables). The statistical analysis focused entirely on birds that managed to become consistently successful. To test the effect of the ‘number of successful trials’ on these response variables (coded durations, log-transformed after adding 0.1 to each data point), we computed mixed models using R (v. 3.2.3 for Mac; packages: lme4, lmerTest, car, and coin) adding subject identity as a random effect. We initially ran LMMs and checked the model residuals for normal distribution (qqplots, Shapiro–Wilk normality test). If the residuals significantly deviated from normality, we conducted GLMMs with a Gaussian distribution and a log-link function. We compared each model to its respective null model using the Akaike information criterion (AIC) to evaluate our approach. For all models presented in the results, the AIC confirmed a better fit of the full model to describe the response variable. Finally, we conducted likelihood ratio tests on the final models. Within-individual comparisons, e.g. the amount of time a subject spent probing with the modified functional tool versus with the non-modified wire, were conducted using exact Wilcoxon signed-rank tests [25]. Sequential Bonferroni corrections were applied in cases of repeated comparisons [26].

## 3. Results

### (a) Bending task

**Figure 3.**
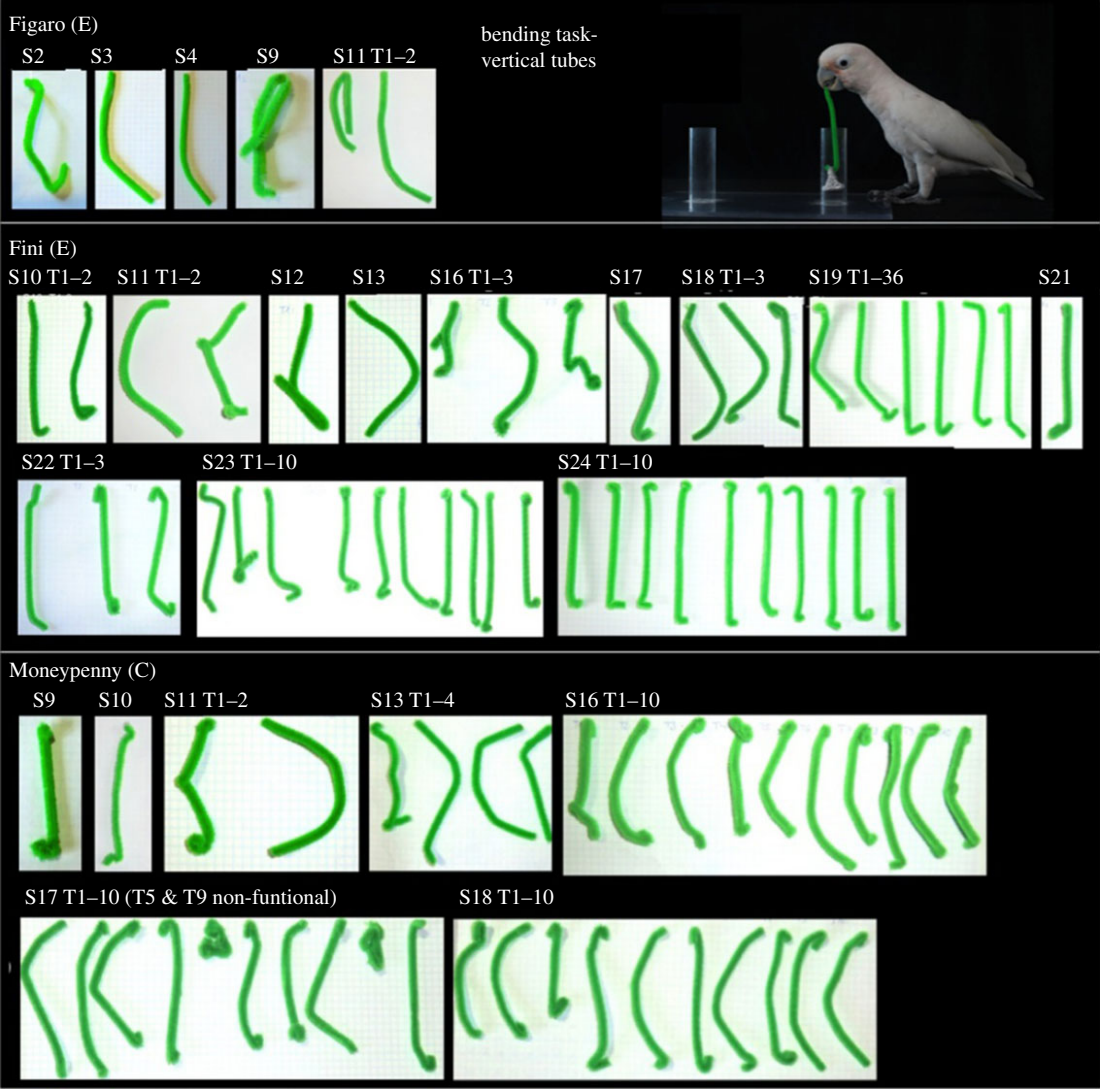
Successful hook-tools build by subjects Figaro, Fini and Moneypenny in the vertical tube condition. S, session number; T, tool number; (C), control group/no experience; (E), experience group; Photograph by Solvin Zankl.

At the beginning of the experiment, all cockatoos received one initial test trial with food being placed in a small bucket inside one of two vertical tubes. A straight piece of pipe cleaner and string were placed alongside the set-up. None of the task-naive birds solved the problem of retrieving the food on the first trial. In the course of the experiment, however, a total of three subjects were able to manufacture hooked tools and to successfully retrieve the food. Two birds (Fini, Figaro) were from the pre-experience group, one bird (Moneypenny) from the control group. Of these, one subject with pre-experience (Fini) and one without (Moneypenny) became consistently successful (figure 3). The third bird (Figaro) succeeded in six occasions of 21 *Phase I* trials in total.

During the first scaffolding step (*Pre-experience A: wire bent to hook, see* figure 2)*,* Figaro was the only bird that used the pre-made hook-tool. He inserted the hook-tool in the correct orientation in 84.4% of trials from the first session of *Phase 2* on and successfully retrieved the basket. Fini successfully repeated *Pre-experience A* after completing the second scaffolding step (*Pre-Experience B: hook inserted in tube, see* figure 2) and used the tool in the correct orientation in 55.6% of trials (20 out of a total of 36 trials; for details see electronic supplementary material, section C). Both subjects turned incorrectly orientated tools around. The remaining subjects failed *Pre-experience A*; most succeeded instantly with the pre-inserted hook-tool in *Pre-experience B* (except two subjects that succeeded from session 2 of *Pre-experience B* on) but, unlike Fini, failed to actively bend a hook when being re-tested with the original problem. Moneypenny was the only subject that successfully bent hook-shaped tools without any pre-experience from session 9 on. She became consistently successful from session 16 on (figure 3). The remaining birds in both groups also initially manipulated and tried to insert or inserted the material a few times but soon lost motivation and failed to produce successful hooks within the time given.

Qualitatively, in the two birds that became consistently successful, hooks improved over time and later tools were bent only at the far distal end at 79° ± 6.5° SE (Fini) and 44.6° ± 9° (Moneypenny; see figure 3). The models (for Fini and Moneypenny combined) showed a significant effect of number of successful trials on ‘time until success’ (LMM, *χ*^2^ = 40.424, d.f.1 = 1, *p* < 0.001), ‘latency from start of the trial until first modification of the wire’ (LMM, *χ*^2^ = 44.738, d.f.1 = 1, *p* < 0.001), ‘duration of probing with the functional end of the modified tool’ (GLMM, *χ*^2^ = 6.531, d.f.1 = 1, *p* = 0.011) and ‘duration of probing with the unmodified wire’ (GLMM, *χ*^2^ = 24.319, d.f.1 = 1, *p* < 0.001). The amount of successful trials had no significant effect on the ‘duration of probing with the modified non-functional tool’ (GLMM, *χ*^2^ = 0.580, d.f.1 = 1, *p* = 0.446). Fini's and Moneypenny's time until success, latency from the start until the first modification of the wire, as well as the duration of probing with the functional modified tool and the unmodified wire decreased over the trials (see electronic supplementary material, section C, c; figures S5 and S6) while the duration of tool crafting remained relatively constant over the trials (see electronic supplementary material, section C, c; LMM: *χ*^2^ = 0.179, d.f.1 = 1, *p* = 0.674). All successful subjects used a variety of bending techniques such as bending the proximate end with the upper over the lower mandible while often holding the wire with one foot, bending the proximate end with the upper over the lower mandible while the distal end was inserted into the tube and (in a few instances) bending of the proximate end over the rim of the tube with the beak or foot while the distal end was inserted (see electronic supplementary material, section C). After hook-bending Fini immediately turned the tool around and used the functional end of the modified tool in order to probe for the basket in 92.7% of successful trials (*n* = 43), Moneypenny in 73.5% (*n* = 36). Furthermore, subjects spent more time probing with the modified functional tool, than with the modified but non-functional tool (Wilcoxon signed-rank test, *Z* = 6.984, *p*_exact_ < 0.001; see electronic supplementary material, section C, c) or with the non-modified wire (*Z* = 7.499, *p*_exact_ < 0.001).

### (b) Unbending task

**Figure 4.**
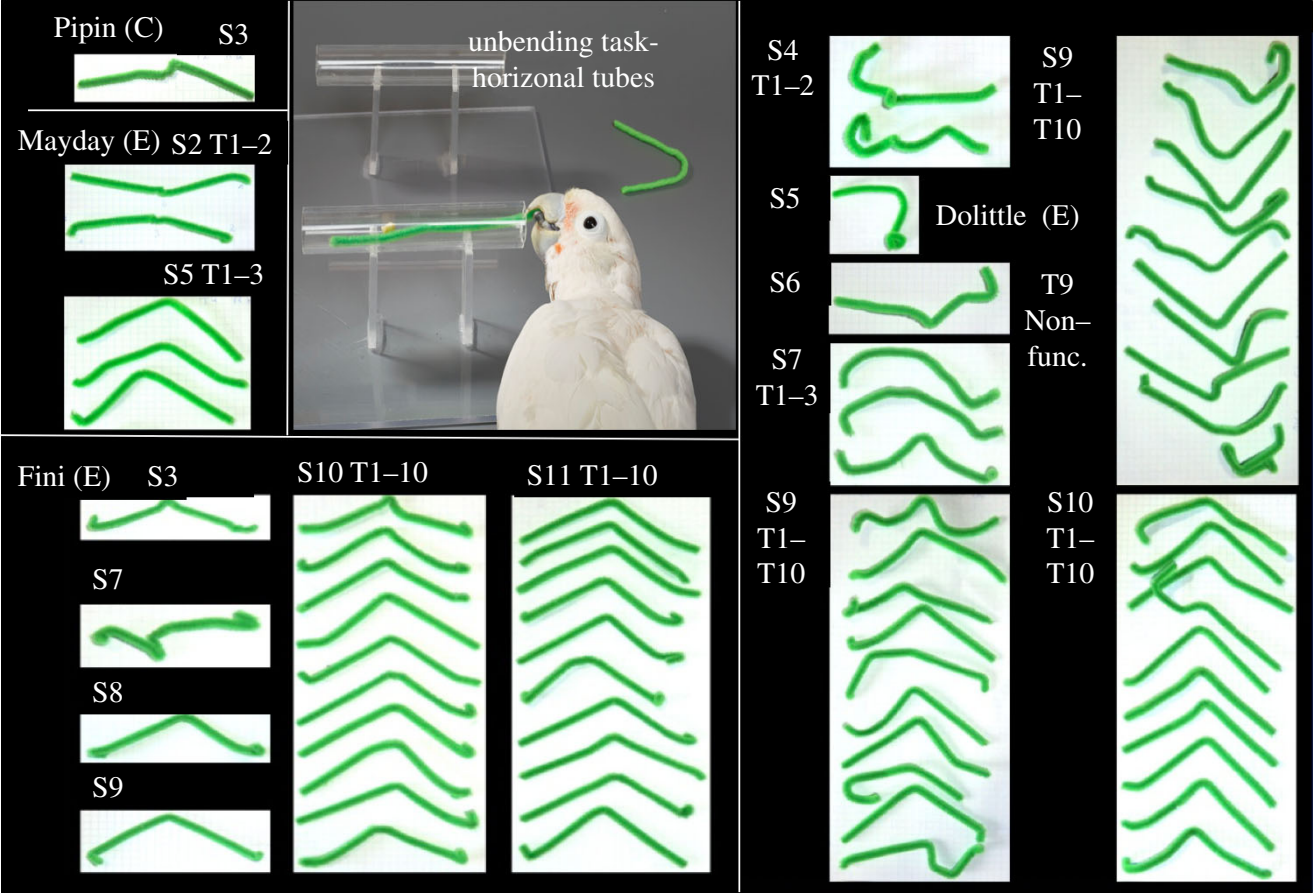
Successful stick-tools build by subjects Pipin, Mayday, Fini and Dolittle in the horizontal tube condition. S, session number; T, tool number; (C), control group/no experience; (E), experience group; Photograph by Bene Croy.

As with vertical tubes, none of the task-naive cockatoos solved the problem of unbending a potential tool (v-shaped wire) for retrieving food from a horizontal tube on the first naive trial. In the course of the experiment, however, a total of four subjects came to succeed in the task (figure 4). Three subjects (Dolittle, Fini, Mayday) had received pre-experience beforehand (*Pre-experience A*: wire straightened out; see figure 2 and electronic supplementary material, figures S1 and S2). Of these, Dolittle and Fini became consistently successful, whereas Mayday was only successful on five occasions (see electronic supplementary material, figure S2). The other subjects of the group with pre-experience (Mup, Muk, Hei, except Figaro) failed to insert the tool during the first step of scaffolding (*Pre-experience A)*; all of them passed the second step (*Pre-experience B*: straight wire inserted in tube, see figure 2), but failed again when being re-tested with the initial problem. One subject of the control group (Pipin) was successful in unbending the wire and retrieving the food without any scaffolding, but he did so in a single test trial only.

The linear mixed models (Dolittle and Fini combined) for the horizontal condition showed a significant effect of number of successful trials on ‘time until success’ (LMM, *χ*^2^ = 52.418, d.f. = 1, *p* < 0.001), ‘latency between start of the trial until the first modification of the wire’ (LMM, *χ*^2^ = 11.876, d.f. = 1, *p* < 0.001), ‘duration of probing with the functional end of the modified tool’ (LMM, *χ*^2^ = 5.896, d.f. = 1, *p* = 0.015), ‘duration of probing with the unmodified wire’ (LMM, *χ*^2^ = 91.344, d.f. = 1, *p* < 0.001), ‘duration of probing with the modified non-functional tool’ (LMM, *χ*^2^ = 100.24, d.f. = 1, *p* < 0.001) and ‘tool crafting time’ (LMM, *χ*^2^ = 11.98, d.f.1 = 1, *p* < 0.001). Fini's and Dolittles's time until success, latency between start and first modification of the wire as well as all three defined categories of probing with the wire decreased in the course of the trials (see electronic supplementary material, section C, d, figures S7 and S8) while tool crafting time remained relatively constant over the trials (see electronic supplementary material, section C, d, figures S7 and S8). Fini, Dolittle and Mayday also used a variety of techniques including unbending with the beak while holding the wire with one foot, unbending with the beak while the wire was partly inserted into the tube and unbending of the wire on the rim of the tube while the wire was inserted (see unbending techniques, electronic supplementary material, section e). In the first few trials Dolittle occasionally unbent the wire by holding one end in his beak while using his foot to straighten the wire. Fini and later Dolittle mainly unbent the wire when it was partly inserted into the tube by forcing it into the tube with the beak. All subjects immediately used the modified functional tool for probing for food. Fini and Dolittle spent more time probing with the modified functional tool than with the unmodified end of the modified tool (Wilcoxon signed-rank test, *Z* = 2.038, *p* = 0.041) or the non-modified wire (Wilcoxon signed-rank test, *Z* = 6.355, *p*_exact_ < 0.001). The wire was typically picked up and manipulated from the beginning of the trial on (mean duration between start and first touch of the wire: Dolittle = 2.3 ± 3.1 s; Fini = 1.1 s ± 0.5 s; Mayday = 2.1 s ± 2.6 s, Pipin = 0.7 s).

## 4. Discussion

Getting back to the first of the two main aims of this experiment, we can hereby confirm that tool-experienced but task-naive Goffin's cockatoos can innovate the crafting of a functional hook from a novel pliant material. They do so despite the apparent lack of predispositions from behavioural routines involving the bending of stick-like structures during foraging or nest building, as it has recently been discussed for corvids [15,16]: three out of a total of 13 cockatoos independently bent straight pieces of wire into hooks and four birds unbent bent pieces of wire; in both conditions, two birds became consistently successful in using the crafted tool for retrieving food. The fact that the tasks were solved by a limited number of birds only and that none of those birds could find the solution in the very first test supports the assumption that Goffin's cockatoos have to individually innovate the solution to the problem. As one cockatoo became consistently successful in both tasks, bending and unbending the wire, it seems to be within the species' capacity to flexibly make different tool types from the same material. Previous studies have already shown that Goffin's cockatoos can make the same tool type from different materials [21].

Compared to corvids the innovation of the first successful tools took longer in the cockatoos: the New Caledonian crow Betty was successful first time in her second trial [1]; after given pre-experience with ready-made hook tools three out of four rooks were successful from the first test trial on, the remaining rook from the fourth trial on [17]. This could indeed be due to species-specific predispositions and adaptations of corvids [15,16], which could facilitate and speed up their innovative process.

Nevertheless, we do not believe that random interactions with the provided materials followed by trial-and-error learning have been sufficient for the cockatoos to succeed within the available time: an entire series of concise actions requiring high levels of motor control had to be applied in a specific order, only the last part of which was rewarded (e.g. only bent the distal part of the wire while keeping the rest of the straight shape intact; the curvature of the hook has to have the right angle; the functional tool needs to be turned and inserted into the tube; pull only when the handle of the basket is hooked; keep pulling until the basket is over the rim). Furthermore, each of the cockatoos tried a variety of different techniques for both bending and unbending, which indicates that subjects did not stick to a learned chain of manipulative actions. Interestingly, and different to corvids, the Goffin's functional hook ‘designs’ seemed to improve over time, with later tools having nearly perfect hooks, bent only at the far distal end and, at least in one bird, at a much steeper angles than the corvid hooks [1,17] (figure 3). Corvid tools were also bent more centred than cockatoo tools, making wider angles better for rooks as they ran out of wire while fishing for the basket. We assume that different hook-making techniques may be the reason for this outcome: Betty bent the distal end of the wire on most occasions by wedging the tip and pulling sideways from the proximal end, levering the wire around the tube or other objects [8] and the rooks bent their hooks over the rim of the tube while the wire was inserted into the tube [17]. While the Goffin's also bent the wire over the rim of the tube in a few instances and used different techniques for fixating the main part of the tool, in the end they mostly bent the hook directly inside their beak.

Cockatoos that became consistently successful showed a learning effect in terms of overall efficiency: the latency to succeed decreased both in the bending as well as in the unbending condition. Interestingly, here, not the time of the crafting of the tool but the time of probing decreased. However, if we look at the actual tools, an improvement of the product of the crafting may have reduced probing time. Successful subjects also spent less time probing with the unmodified tool than for probing with the modified functional tool, the orientation of the manufactured functional tools was mostly correct and the distractor material was hardly touched.

The second main aim of this study was to identify the effect of individual pre-experience with hooks for potential task success. Pre-experiencing bent or unbent wire pieces seemed to help some individuals to develop their crafting skills. However, receiving pre-experience appeared not to be necessary to succeed in either task. One bird of the three birds that did become efficient hook-benders was from the control group and did not receive any scaffolding. This strongly indicates that the ability to innovate a hook tool without having experienced a ready-made hook before seems to lie within the species' capacity. From the start, Moneypenny (control group) manipulated mainly the wire (see electronic supplementary material, section C, d), thereby producing hook-like shapes out of the wire in seven out of eight sessions before succeeding for the first time in session 9. It is unclear whether the animal required a mental representation of the tool type. As the string was hardly touched, it is plausible that she had determined that the tool had to be solid rather than flimsy. Furthermore, as she altered the wire after fishing attempts with the original material did not lead to success, she seems to have apprehended that the current shape was non-sufficient. Alternatively, she could have initially started to bend the material out of frustration. Nevertheless, while she did try out different modifications, as mentioned before, those were unlikely to have been entirely random. For Fini (test group) it seems that *pre-experience B* was essential for success, since we could observe a strong increase in wire-manipulation time (see electronic supplementary material, section C, d) and she started modifying and inserting the wire after receiving this pre-experience.

When looking at the initial orientation of the ready-made hooks during scaffolding steps, Figaro was highly successful early on. Fini, by contrast, seemed to use trial and error learning to accomplish the correct tool orientation but did show motor flexibility: incorrect inserted tools were immediately turned around without dropping them in between (a change in orientation of the hook after dropping a tool and picking it up again could be accidental). This effect was stronger in trials with self-crafted hooks. Here subjects also probed the tube longer with the modified tools.

In the unbending condition only one bird in the control group succeeded a single time versus three birds in the test group, two of which became consistently successful. It is therefore possible that experience had a bigger effect on success on this task. Nevertheless, as birds unbent the wire mainly inside the tube while forcing through the opening it is likely that experiencing a functional tool merely increased their motivation to insert available solid materials into the tube.

Taken together, some cockatoos not only actively invented solutions to the hook bending/unbending problem, but seemed to acquire an appreciation of the corresponding functional versus non-functional task properties. Our results also suggest that hook bending from pliant material in this species does not require hereditary predispositions from specialized tool use/manufacture or nest building, but may arise from more general modes of cognitive processing. Although those modes cannot yet be fully confirmed, they most likely entail high behavioural flexibility, sensorimotor control, fast individual learning and a propensity for haptic exploration and object combination. Referent to the current reproach of the corvid studies [16] we would like to emphasize that innovative problem-solving does not require the complete exclusion of pre-existing behaviours [27]. In contrary we believe that innovation almost always requires some recruitment of pre-existing skills. In the cockatoos those are not necessarily entire behavioural sequences used to make a similar product but if we break their behaviour into smaller components, like grading the lower against the upper mandible, we have a behavioural element that can be used for extractive foraging and to refine the shape of a hook. Considering the famous example of the New Caledonian crow Betty [1] from this point of view, we must acknowledge that her hereditary predispositions likely were of advantage for this particular task type; yet, she was able to combine her predisposition with her experience and creative cognition in order to solve a novel problem including a novel material. This does not devaluate her performance from being innovative but brings us closer to understanding the ingredients of seemingly sophisticated behavioural solutions to tool-use problems.

## Supplementary Material

Supplementary Information Laumer et al. 2017

## Supplementary Material

Dataset Laumer et al. 2017

## Data accessibility

The dataset supporting this article has been uploaded as part of the electronic supplementary material.
