# Supplementary material for: Can hook-bending be let off the hook? Bending/unbending of pliant tools by cockatoos
Source: Proc Biol Sci. 2017 Sep 6;284(1862):20171026. doi: 10.1098/rspb.2017.1026 (PMC5597828; doi:10.1098/rspb.2017.1026)
Supplement: Supplementary Information Laumer et al. 2017 [file rspb20171026supp1.docx]

**Supplementary Information**

**A) Subject information**

**Table S1** Names, division into two testing groups (E= experience group; C= control group), sex and age of the 13 Goffin’s cockatoos.

| **Group** | **Name** | **Sex** | **Hatched** |
| --- | --- | --- | --- |
| E | Dolittle | male | 2011 |
|  | Figaro | male | 2007 |
|  | Muppet | male | 2010 |
|  | Muki | male | 2011 |
|  | Fini | female | 2007 |
|  | Mayday | female | 2011 |
|  | Heidi | female | 2010 |
| C | Konrad | male | 2010 |
|  | Zozo | male | 2010 |
|  | Pipin | male | 2008 |
|  | Kiwi | male | 2010 |
|  | Moneypenny | female | 2010 |
|  | Olympia | female | 2010 |

*Housing*

Subjects are permanently housed in a large enriched aviary with indoor and outdoor area (indoors: 45m^2^ ground space, 3 to 6 m high; outdoors: 150m^2^, 3 to 4,5m high). The indoor area is kept at 20 C° during winter time and electric UV light is switched automatically at 12:12 hours light-dark cycles. Food (Cardi-seed mix by Animalexperts.at supplemented with dried fruit and vitamins; diverse sorts of fresh fruits and fresh and cooked vegetables, Soy Jogurt), mineral sources and fresh drinking water was available at libitum. Special treats such as nuts were only offered as food rewards during experimental routines. All birds were hand-raised and are marked with coloured leg bands for identification. All animals participated on a voluntary basis and were never food or water deprived.

*Experimental histories*

All subjects had experience in inserting compact tools and in inserting stick tools in an apparatus in order to poke a reward off a platform ^22^. Four subjects (Figaro, Kiwi, Dolittle & Pipin) had experience retrieving a reward from behind a wire mesh using a stick tool ^19, 20^.

**B) Supplementary Procedures:**

*1) Habituation phase*

*Habituation to apparatus*

Subjects received two sessions of 20 trials of each condition: In the vertical condition subjects had to retrieve a basket (made of aluminium foil; baited with a piece of cashew nut) out of a short vertical tube by using their beak, in the horizontal condition the reward rested in a short horizontal tube and could be reached with the beak. The position of the reward was semi-randomized across sessions. Irrespectively of their group affiliation half of the subjects received the vertical condition first, the other half were tested first in the horizontal condition.

*Habituation to novel material*

To give subjects the information that the wire but not the string retains its form after bending, subjects observed the experimenter warp a wire and a string around a pen a total of nine times in random order on two consecutive days. Afterwards subjects received a total of two sessions of 15 minutes to familiarize with the wire and string.

*Analysis*

**Table S2** List of variables measured in analysis

| ***Variables measured*** |  |  |  |  |  |  |  |
| --- | --- | --- | --- | --- | --- | --- | --- |
| Time until success (excluding time when subject was not interacting with material/apparatus) | | | | | | |  |
| Duration of probing with unmodified wire (probing is defined as one end of wire is inserted into the tube) | | | | | | |  |
| Duration of probing with modified non-functional wire | | | |  |  |  |  |
| Duration of probing with modified functional tool | | | |  |  |  |  |
| Latency between start of the trial and first touch of the wire | | | | |  |  |  |
| Latency between start of the trial and first modification of the wire | | | | |  |  |  |
| Tool crafting time (duration spent modifying the wire) | | | | |  |  |  |
| Proximal, middle or distal modification | | |  |  |  |  |  |
| Place of modification (at apparatus or elsewhere) | | | |  |  |  |  |
| Functional or non-functional end of modified tool first used to probe | | | | |  |  |  |
| Technique used to modify wire | |  |  |  |  |  |  |
| Wire turned around and how often | | |  |  |  |  |  |
| Final shape of tool | |  |  |  |  |  |  |
| String touched |  |  |  |  |  |  |  |
| Duration of string manipulation | |  |  |  |  |  |  |
| String inserted |  |  |  |  |  |  |  |
| Duration of string insertion | |  |  |  |  |  |  |
| String or wire inserted in unbaited tube | | |  |  |  |  |  |
| Duration of insertion in unbaited tube | | |  |  |  |  |  |

**C) Supplementary results**

1. ***Progress of Success***

**
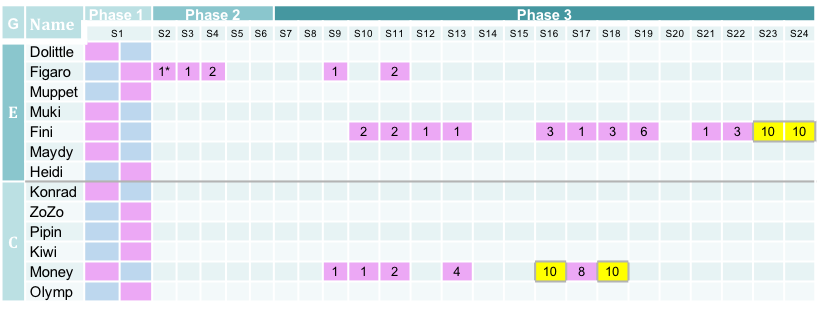
**


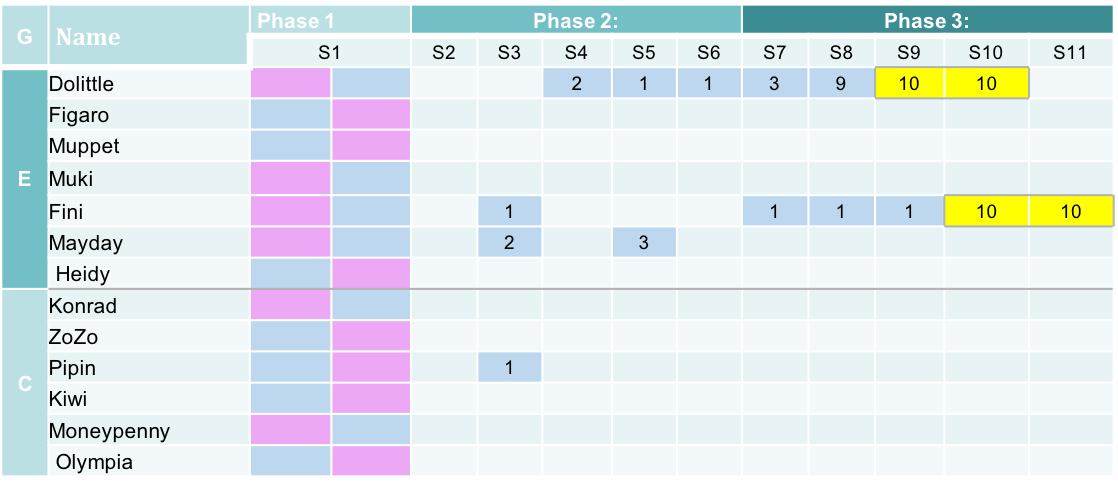


**Figures S1& S2.** Overview over the testing procedure and results of all *Phase I* trials of the bending (in pink) and unbending task (in blue). Phase 1 indicates the colour condition that subjects started with. The remaining results of *Phase I* in the bending task are depicted in Figure S1 (above) and in the unbending task in Figure S2 (below). Unfilled cells indicate unsuccessful sessions, numbers indicate the number of successful *Phase I* trials within the respective session. Sessions in which subjects were consistently successful in all *Phase I* trials per session are marked in yellow. Numbers with asterisks indicate successful *Phase I* trials by using the unmodified straight wire.

In the vertical condition Figaro (Group E) was the only bird that managed to continuously insert the pre-bent wire and successfully retrieved the basket from the first session of *Phase II* on. Consequently he was the only bird that was tested in *Phase I* (naïve phase) during Pre-experience A (=*Phase II).* Dolittle, Fini, Mayday and Heidi continuously successfully retrieved the basket from the first session of Pre-Experience B (=*Phase III*) on, Muppet and Muki from the second session on. After completing ten trials of *Phase II or Phase III* during one session, subjects received up to ten *Phase I* trials, depending on task success. For details on experimental procedures see methods.

In the horizontal condition Figaro, Fini and Mayday (group E) successfully inserted the given straight wire and retrieved the reward from the first session of *Phase II* on, Dolittle from the second session on. Muppet, Muki and Heidi continuously retrieved the reward from the first session of Pre-Experience B (=*Phase III*) on. After completing ten trials of *Phase II or Phase III* during one session, subjects received up to ten *Phase I* trials, depending on task success.

***b) Correct orientated insertions of pre-made hook tools in the pre-experience phase of the vertical condition***

**
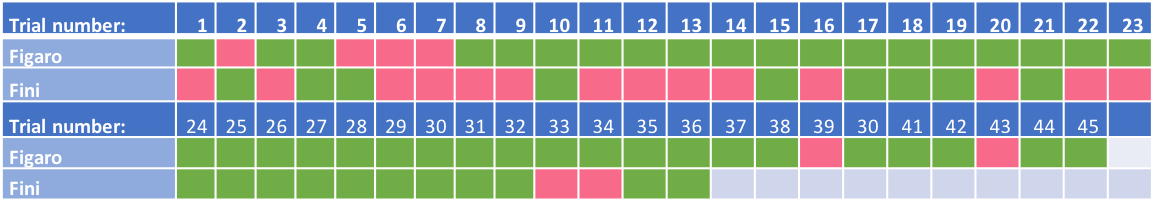
**

**Figure S3** Correct orientated insertions of pre-made hook tools (1/green= correct orientated insertion, 0/pink= incorrect orientated). Only the first insertion of each trial is depicted here. Figaro inserted the hook-tool in correct orientation in 84.4% of trials (38 out of a total of 45 trials), Fini in 55.6% of trials (20 out of a total of 36 trials).

***c) Descriptive results of successful trials in the hook-bending task***

**Fig. S4** Results of Figaro´s successful trials in the vertical condition (total number of successful trials = 6 trials). Note that if more than one trial was successful within one session, the trials are depicted here in ascending order on the x-axis with the same session labelling.

**Fig. S5** Results of Fini´s successful trials in the vertical condition (total number of successful trials = 43 trials). Note that if more than one trial was successful within one session, the trials are depicted here in ascending order on the x-axis with the same session labelling.

**Fig. S6** Results of Moneypenny´s successful trials in the vertical condition (total number of successful trials = 36 trials). Note that if more than one trial was successful within one session, the trials are depicted here in ascending order on the x-axis with the same session labelling.

*Additional results*

The wire was typically picked up and manipulated from the beginning of the trial on (mean duration between start and first touch of the wire: Figaro=1.7s±0.5; Fini=1.2s±0.6; Moneypenny=10.5s±54.3). The three birds rarely manipulated the distractor material (mean manipulation time of the string per trial: Figaro=14.2s±27.9 sec; Fini=0.1s±0.4 sec; Moneypenny=0.7s±1.7 sec) and rarely inserted the wire into the control tube that did not contain any food (mean duration of wire insertion in the control tube per trial: Figaro=0.6s±1.2 sec; Fini=0.1s±0.5 sec; Moneypenny=3.6s±10.2 sec).

**d) Descriptive results of successful trials in the unbending task**

**Fig. S7** Results of Dolittle´s successful trials in the horizontal condition (total number of successful trials = 35 trials). Note that if more than one trial was successful within one session, the trials are depicted here in ascending order on the x-axis with the same session labelling.

**Fig. S8** Results of Fini´s successful trials in the horizontal condition (total number of successful trials = 24 trials). Note that if more than one trial was successful within one session, the trials are depicted here in ascending order on the x-axis with the same session labelling.

**Fig. S9** Results of Mayday´s successful trials in the horizontal condition (total number of successful trials = 5 trials). Note that if more than one trial was successful within one session, the trials are depicted here in ascending order on the x-axis with the same session labelling.

*Additional results*

The birds rarely manipulated the distractor material (mean manipulation time of the string per trial: Dolittle=6.2±21.6 sec; Fini=0.1s±0.2 sec; Mayday=9.3s±16.1 sec, Pipin=0 ses) and rarely inserted the wire into the control tube (mean duration of wire insertion in the control tube per trial: Dolittle=0.9±2.8 sec; Fini=0 sec; Mayday=2.6s±5.3 sec, Pipin=12.5 sec).

***e) Descriptive results of unsuccessful trials in the hook-bending task***

**Fig. S10** Duration of string and wire manipulation of subject Moneypenny (control group) before being successful.

**Fig. S11** Duration of string and wire manipulation of subject Fini (test group) before being successful. Subject received pre-experience 1 from session 2 to 6 and pre-experience 2 from session 7 on.

***f) Bending and unbending techniques***

**
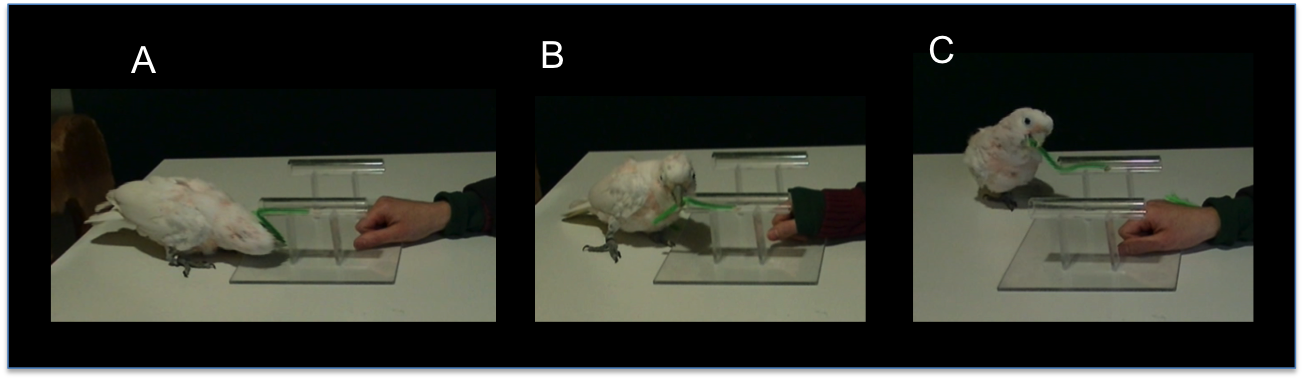
**

**Fig. S12** Different unbending techniques.

**
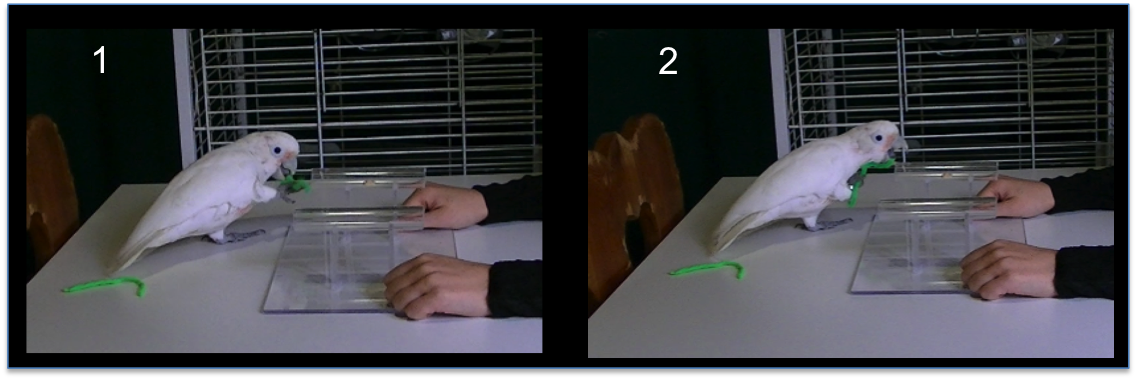
**

**Fig. S13** Unbending of a previously bent ball-shaped piece of wire into a straight shape.

***g) Videos of successful trials in the hook bending and unbending task***

**Video S1** Video of subject Fini in the vertical and horizontal condition.
